# Residual Fire Resistance Testing of Basalt- and Hybrid-FRP Reinforced Concrete Beams

**DOI:** 10.3390/ma15041509

**Published:** 2022-02-17

**Authors:** Kostiantyn Protchenko

**Affiliations:** Department of Civil Engineering, Warsaw University of Technology, 16 Armii Ludowej Av., 00-637 Warsaw, Poland; k.protchenko@bimplatform.pl

**Keywords:** fiber reinforced polymers FRP, FRP bars, FRP reinforced concrete members, fire resistance of FRP-RC beams, basalt FRP, hybrid FRP

## Abstract

The fire resistance of fiber-reinforced polymer reinforced concrete (FRP-RC) elements depends on the temperature performance of the original concrete member, the fire scenario, and FRP reinforcement behavior. In this study, fire resistance tests are described, along with the characteristics obtained during and after applying elevated temperatures, simulating the effects of fire. The tested beams were reinforced with basalt (BFRP) bars and with a hybrid composite of carbon fibers and basalt fibers (HFRP) bars. Fire tests were performed on full-scale beams, in which the midsections of the beams were heated from below (tension zone) and from the sides for two hours, after which the beams were cooled and subjected to flexural testing. BFRP-RC beams failed before the heating time was completed; the best failure was associated with a BFRP reinforced beam that failed approximately 108 min after heating. Contrary to the BFRP-RC samples, HFRP-RC beams were capable of resisting exposure to elevated temperatures for two hours, but showed a 70% reduction in strength capacity when compared to non-heated reference beams. According to the author, the higher resistance of HFRP-RC beams was the result of the thermal expansion coefficient of carbon fibers employed in HFRP, which “prestresses” the beams and enables smaller deflections. The preliminary findings of this study can increase the feasibility of using FRP materials for engineering purposes.

## 1. Introduction

First developed in the 1970s, fiber-reinforced polymers (FRPs) have attracted the attention of architects and civil engineers because of their superior characteristics, such as their high strength, light weight, corrosion resistance, and transparency to magnetic fields. Many studies have been conducted on the use of FRP bars as internal reinforcements in reinforced concrete (RC) structures [1,2,3,4,5]. FRP bars possess unique properties determined by their matrix and fibers; therefore, it is essential to examine these properties and understand their behavior to develop more precise, safer, and cost-effective designs [6,7,8].

The development of FRP technologies has led to the introduction of new types of FRP bars, for which it will be possible to adjust the sustainability and cost. In spite of the availability of many commercial types of FRP bars with adequate properties, they are usually either less expensive and less durable than steel, or more expensive but with better properties.

For this study, BFRP and hybrid (HFRP) bars were used as internal reinforcements. The BFRP bars have great potential as cost-effective and efficient reinforcement materials for concrete structures. However, BFRP bars exhibit a relatively low modulus of elasticity and linear elastic behavior until failure; the flexural behavior of BFRP-RC members is not ductile, as it is in steel-reinforced beams [9,10,11,12]. The HFRP bars used in this work were constructed from basalt and carbon fibers. Carbon fibers are suitable from the standpoint of sustainability and have superior mechanical properties; however, their production is very energy-intensive, making them financially and environmentally expensive [13,14,15]. In the process of producing HFRP (HC/BFRP) bars, some of the basalt roving was replaced with carbon roving to achieve better mechanical properties than CFRP bars but at a much more lower cost. HFRP bars are manufactured similarly to BFRP bars and other types of commercially available FRP bars.

Fire resistance is one of the greatest concerns engineers and researchers face when using FRP bars, since the behavior of FRP reinforcements and FRP-RC structures during exposure to heat should be carefully taken into account before implementing these materials [16]. RC structures may sustain significant damage from fire and elevated temperatures; therefore, reliable and accurate estimates of potential risks are imperative [17,18]. On the other hand, different standards describe the behavior of FRP reinforcements during fire exposure, such as the Canadian Code CAN/CSA S806-12 Annex R [19] and the American Concrete Institution ACI 440.1R-15 [20]. However, neither basalt FRP (BFRP) nor hybrid FRP (HFRP) is referred to in the standards. Currently, FRP reinforcement is not widely used in RC structures because of several factors, including their unknown fire resistance, which limits their use to situations where fire resistance is not relevant [21]. FRP reinforced concrete elements and structures have been studied under elevated temperatures by numerous researchers. Robert and Benmokrane [22] examined GFRP bars that were exposed to various elevated temperatures ranging between 23 and 315 °C. At temperatures above the glass transition temperature of the polymer matrix, the mechanical characteristics of the composites were diminished. At approximately 315 °C, bars lose about 53% of their primary tensile strength. The residual tensile properties of GFRP bars were studied by Ellis et al. [23]. The bars were subjected to elevated temperatures (up to 400 °C) and the tensile tests were performed after cooling. As a result, the GFRP bars after cooling retained 83% of their tensile strength. Wang and Zha [24] investigated the mechanical and physical characteristics of GFRP and CFRP bars at elevated temperatures. The stress-to-strain relationships of FRP bars from the obtained results displayed almost linear behavior until failure at elevated temperatures. The modulus of elasticity of FRP bars remained constant between 300 and 400 °C, but as soon as this temperature was exceeded, the modulus declined rapidly. When exposed to temperatures exceeding 500 °C, the tensile strength of the bars declined almost linearly. Hamad et al. [25] studied the effects of elevated temperatures on the mechanical characteristics of various FRP bars and reported that at a critical temperature of 325 °C, there was up to 55% loss in their tensile strength.

As can be seen from the previous studies, the application of FRP-RC elements has increased over the past decades, and it can be viewed as a substitute for traditional steel reinforcement in concrete structures [26]. Hence, FRP reinforcement in RC structures should meet the stringent fire-resistance requirements prescribed in building codes. Researchers should investigate how FRP reinforcement can meet the criteria for fire resistance. A limited amount of information is available regarding the behavior of structural elements reinforced with FRP in fire conditions.

As Weber [27] pointed out, it is important to distinguish between the two different temperature limits, one relating to the decrease in tensile strength of the bars and the other to the deterioration of the bond strength. Tensile tests at temperatures of 400–500 °C showed that the strength decreased for bars by 30% to 80%. In addition, the bond strength between concrete and FRP significantly decreases as the glass transition temperature (T_g_ ≅ 180 °C). Using carbonate aggregate concrete and a thicker concrete cover, Kodur et al. [28] were able to achieve greater fire resistance for FRP-RC slabs. The degradation of reinforcement bonds and the applied load have been identified as the main factors that contribute to the fire endurance of RC slabs. In testing concrete beams reinforced with GFRP bars covered with concrete measuring approximately 70 mm thick, Abassi and Hogg [29] found that the beams can survive fire for more than 90 min after being exposed to elevated temperatures. Consequently, it was recommended that concrete covers for GFRP-RC beams be at least 70 mm thick to ensure fire resistance. However, this recommendation was not accepted as a standard due to economic and practical reasons. The parametric analysis [30] by Saafi found that FRP temperatures decreased as the concrete cover increased, while FRP-RC beams displayed significant degradation in terms of shear and flexural strength. The minimum concrete cover value for fire resistance for FRP-RC elements according to his study is 64 mm.

Few studies have examined the residual characteristics of FRP composites or the post-fire behavior of FRP-RC structures. In GFRP materials, certain degrees of the original elastic modulus and strength can be recovered after exposure to temperatures between glass transition and decomposition. This can be ascribed to the reversibility of the glass transition. Bai et al. [31] conducted dual-run dynamic mechanical analysis (DMA) tests on glass polyester laminate from 0 to 200 °C (higher than the glass transition temperature T_g_ but below the decomposition temperature T_d_). Once the sample was heated to 200 °C, the elastic modulus of the sample dropped to about 24% of its initial value. Alsalihi evaluated the residual mechanical properties of GFRP bars post-heating in his study [32], in which the bars were heated to 250 °C and maintained for 0–30 min. As shown in previous studies [33,34], FRP materials exhibit diminished mechanical properties due to delamination of constituents caused by decomposition of the resin.

Bai et al. [35] analyzed the response of GFRP slab specimens exposed to an ISO-834 fire test (which consists of increasing the temperature to 1100 °C over 180 min). Four-point loading was applied to two samples prior to, during, and after fire exposure. Both specimens lost nearly one-third of their flexural stiffness after they were exposed to fire, but almost half was recovered when they were cooled to ambient temperatures. High temperatures reduce the bonds between FRP bars and concrete, according to the data available [36]; however, little information is available on whether the bonds can be restored once the specimen has been cooled. In order to improve the knowledge of the structural responses of FRP-RC elements after and during exposure to fire and elevated temperature conditions, more comprehensive and practical research should be conducted.

The current study experimentally examines the post-fire performance of RC beams reinforced with BFRP and HFRP bars after being exposed to elevated temperatures to determine their residual strength.

## 2. Novelty and Significance of the Work

A lack of information on the residual characteristics of FRP-RC flexural elements makes it difficult to develop analytical models that are accurate enough to predict the structural behavior of such members and to develop corresponding design provisions. In this study, residual tests were performed on FRP-RC beams that were not loaded during heating in order to analyze how FRP bars respond after they have been heated. The beams were subjected to specific fire actions, with the mid-sections of beams gradually heated from below (tension zone) and from the sides. The author examined the behavior of FRP-RC beams post-fire in a previous study [37]. However, the number of samples was insufficient to confirm the trends in the behavior of the elements (one sample for each type); therefore, three samples for each type with the same characteristics will be discussed in this study. Moreover, based on the referenced information [28,29,30], it was decided to increase the beam dimensions (to achieve a bigger concrete cover from the bottom, the most heated place) and to extend the heating time from one hour to two hours. In addition, the current study focuses on beams reinforced with BFRP and HFRP; hybridization allows engineers an added level of flexibility in selecting compositions with the desired stiffness, strength, dimensional stability, energy absorption, failure strain, corrosion resistance, cost, and other required characteristics.

## 3. Materials and Experimental Program

### 3.1. Decription of Experimental Program

This experimental program consists of designing and constructing 12 full-scale FRP-RC beams without any fire protection. A residual post-fire behavior analysis was conducted for three simply supported beams reinforced with BFRP bars (set 1.1) and for three beams reinforced with HFRP bars (set 1.2). A comparison of the strength properties of the three beams for each type was made against the reference beams (sets 2.1-ref and 2.2-ref).

In order to perform residual testing of FRP-RC beams, the following steps were taken:

(1) To simulate realistic situations, i.e., cracks appearing, beams from set 1.1 and set 1.2 were preliminary loaded up to 50% of their ultimate strength capacity and then unloaded;
(2) The middle section of unloaded, cracked beams was placed inside a furnace so that the temperature could be applied from below and from the sides;
(3) The beams were heated according to the official norms [38] and a standard heating curve ISO-834 (1999) [39] (to initiate the temperatures representative of fire temperatures);
(4) The beams went through a cooling phase for approximately 24 h;
(5) After the cooling phase, the strength of the beams was determined by the four-point flexural test.

Both sets 2.1-ref and 2.2-ref of beams were tested with four-point flexural tests without preliminary loading and were not exposed to high temperatures. In addition, the results were compared with those from previous studies. The previous study results (set 1-prev and set 2-prev-ref) were mainly used to analyze the effects of the heating time and concrete cover on the beam strength and method of destruction. It is worth noting that the reference specimens used for this and previous studies were not preloaded or exposed to fire. As a result of the initial loads used in the fire samples, the samples had a relatively lower load-bearing capacity compared to the reference samples. Despite this, the preloaded and fire-subjected samples had an appropriate level of post-fire resistance.

In Table 1 the general procedure is outlined with regard to the considered sets, while in Table 2 descriptions of the specimens used in current and previous studies are detailed.

### 3.2. Materials

#### 3.2.1. Concrete

For both studies, the concrete mixture used was the typical C40/45 mix, with ordinary Portland cement CEM III/A (Castorama, Warsaw, Poland), ash, and crushed stone (silica) with a nominal maximum size of 16 mm.

Before the beams were placed on the testing frame, they were cured in the lab for 28 days. For confirmation of the concrete class, 100 mm cube specimens of the same concrete mixture were tested for compressive strength 28 days after mixture preparation, in accordance with PN-EN 12390-3 [40]. The mechanical properties of the concrete used for the specimens in the current study and previous study are shown in Table 3. 

#### 3.2.2. Reinforcement

The beams in the tension zone were reinforced with two types of reinforcement, BFRP and HFRP bars. In the context of this work, the term hybridization might be understood as the physical combination of different fibers embedded in epoxy resin. The HC/BFRP (HFRP) bars were made by embedding carbon fibers and basalt fibers in epoxy resin. Due to the similar strain parameters of basalt and carbon fibers of low strength (LS), different volume fractions of carbon and basalt (C/B) were considered and selected.

The effect of the ratio of carbon to basalt fibers and the influence of their location on the mechanical properties of hybrid composites are discussed in previous companion papers [41,42,43,44], along with a detailed and extended description of the characteristics of the bars and their configurations. 

Several technological challenges were encountered while placing carbon fibers in the near-surface region, including increased heterogeneity at fiber locations and local scorching of bars caused by temperature changes. Thus, carbon fibers are most effective in the core region of HFRP bars, where a carbon/basalt ratio of 1:4 is assumed (i.e., 16% carbon fibers, 64% basalt fibers, and 20% epoxy resin). 

As a result of the tensile tests of BFRP and HFRP bars, Table 4 shows the mean values for the maximum strength, *F_u_*; limit stress, *f_u_*; modulus of elasticity, *E*_1_; and limit strain, *ε_u_*. Five samples per type were analyzed to identify the mean values. During previous studies, i.e., sets 1-prev and set 2-prev-ref, the same bars were used.

## 4. Test Setup and Specimen Dimensions

All specimens in the current study had the same dimensions: 140 mm wide, 280 mm high, and 3220 mm long. All specimens had a clear cover measuring 60 mm from the bottom and 40 mm from the other sides.

All beams were reinforced at the top with BFRP bars (8 mm diameter) as longitudinal reinforcement and stirrups made of BFRP bars (6 mm diameter) as shear reinforcement. For mid-sections of beams, the stirrup spacing was assumed to be 100 mm, and no stirrups were present. The lack of stirrups in the middle part of the beams allows one to disregard the effects of other bars in the location of heating and concentrate on the performance of different types of FRP bars in the tensile zone. On the other hand, shear forces are negligible in the middle of the beams. Stirrups in this case are used to provide stability to the longitudinal reinforcement of the beam (mainly to prevent buckling of the upper longitudinal bars due to compression). In the current research, the lower-tension longitudinal reinforcement in the middle section of the beam was stabilized with spacers.

**Figure 1:**
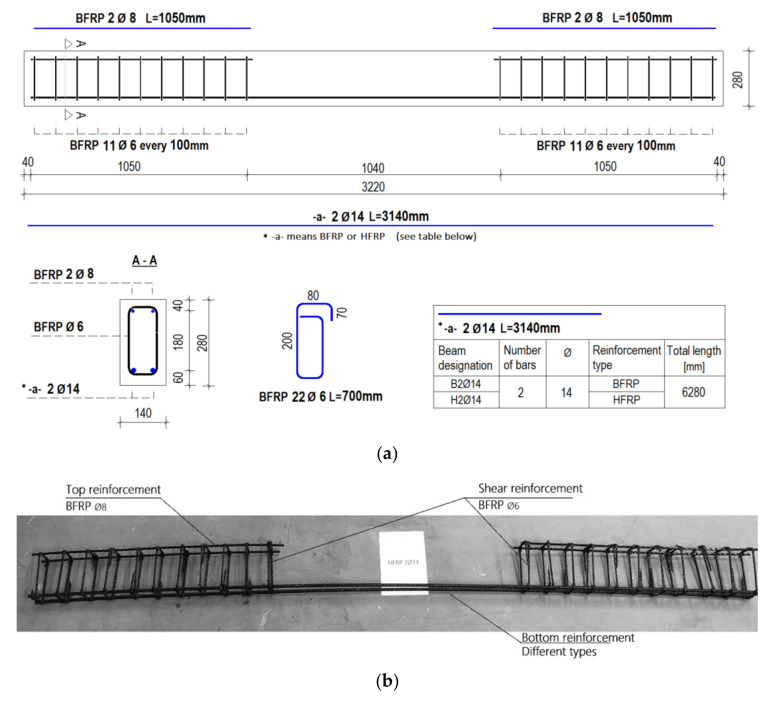
Reinforcement scheme of the tested specimens: (**a**) schematic details of different configurations; (**b**) example of reinforcement configuration (set 1.2.—sample H2Ø14).

Figure 1a shows a scheme of the tested specimens with a variable bottom reinforcement (tension zone). Figure 1b shows an example of a reinforcement configuration (sample H2Ø14) to provide additional information.

**Figure 2:**
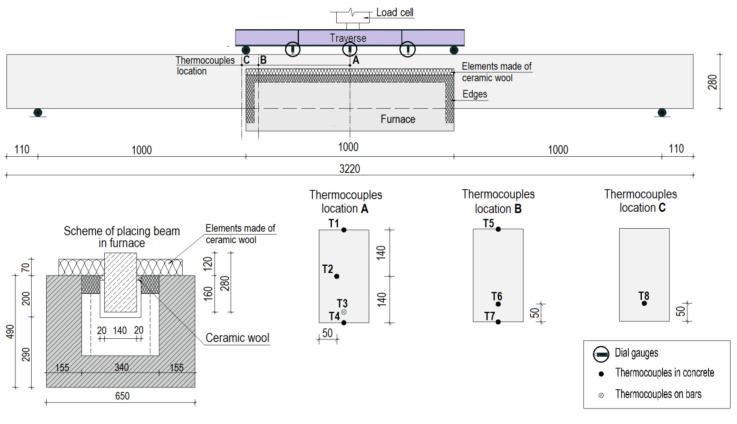
Test setup description.

In order to monitor the temperature during the fire exposure, each specimen was instrumented with type K thermocouples (till 1200 °C) inside the specimens at different locations. For each beam, eight thermocouples were embedded at different depths. Seven thermocouples were embedded in the concrete, while thermocouple T3 was embedded on the surface of a bar. On the top faces of the beams, three dial gauges were applied to measure the deflections in the midsection. In Figure 2, the locations of thermocouples and dial gauges and a description of the test setup are shown.

**Figure 3:**
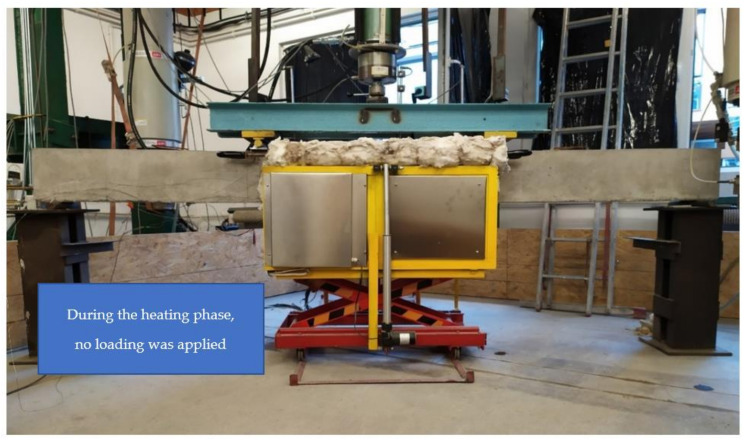
Test setup during the heating phase (set 1.1—sample B2Ø14).

The beams from set 1.1 and set 1.2 were loaded up to 50% of their ultimate strength load (as outlined in Table 2) and then unloaded before the temperature was applied. The unloaded beams were placed in the furnace in such a way that the middle part of the beam, at approximately one-third of its length, was heated. During the heating phase, specimens were heated below and from the sides for two hours. Figure 3 illustrates the test setup during the heating phase. 

The beams were heated according to the norms [38], and a standard heating curve from ISO-834 (1999) [39] was applied (to initiate the fire temperature), which is represented by the following formula:

T_ISO_ = T_0_ + 345 × log(8t + 1), (1)

where T_ISO_ is the temperature (°C), T_0_ is the room temperature (assumed to be 20 °C), and t is the time (min).

Set 1.1 and set 1.2 beams were allowed to cool for approximately 24 h before their flexural strength was tested in four-point bending tests. Set 2.1-ref and set 2.2-ref beams were subjected to four-point bending tests only.

## 5. Results and Discussion

The conducted tests can be used to examine the effects of elevated temperatures on FRP-RC beams, as well as their thermal behavior when subjected to those temperatures. In this study, the results are compared to reference beams and previous results involving slightly different beam dimensions and a one-hour heating period.

### 5.1. Destruction of Samples

**Figure 4:**
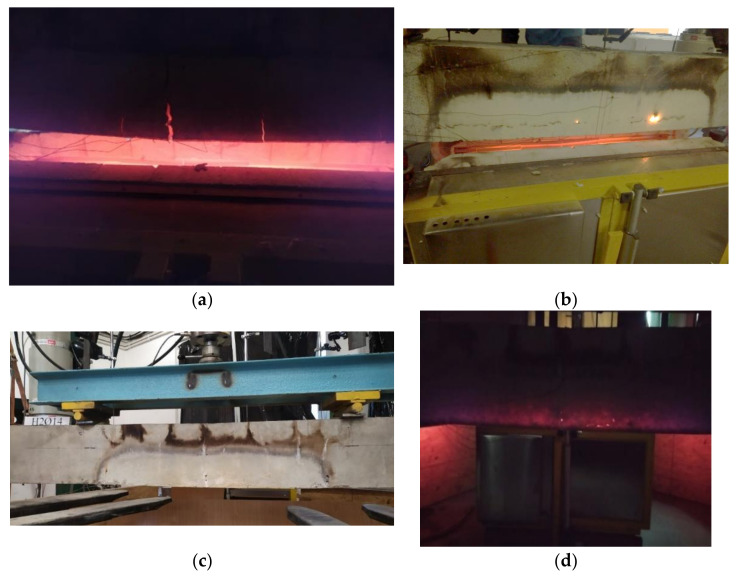
Samples just after being taken out of furnace: (**a**) set 1.1 B2Ø14; (**b**) set 1-prev B2Ø14; (**c**) set 1.2. H2Ø14; (**d**) set 1.2. H2Ø14, no lights.

The first important point to note is that not all beams exhibited the ability to withstand elevated temperatures for a period of two hours. Three BFRP-RC beams (set 1.1) failed during the heating phase, as opposed to the samples from the previous study, set1-prev. All HFRP beams (set 1.2) were able to resist temperatures and allowed to cool. In Figure 4, a representation is shown of some of the samples immediately after they were removed from the furnace.

As in the previous study, all samples subjected to residual tests were destroyed as a result of reinforcement failure, whereas the reference beams were destroyed as a result of concrete crushing. This indicates that elevated temperatures adversely affect the strength of FRP bars.

**Figure 5:**
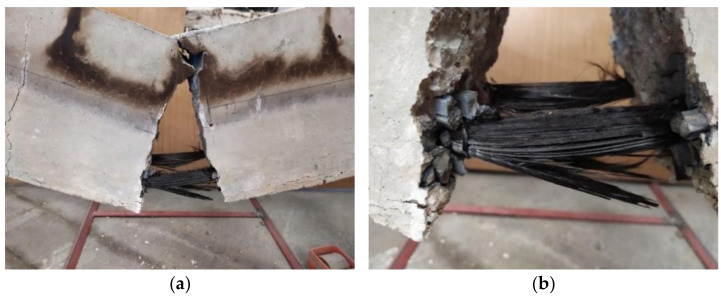
Destruction of specimen B2Ø14: (**a**) non-magnified image; (**b**) magnified image.

The BFRP-RC beams were destroyed due to failure of the reinforcement during the heating period; despite their increased concrete cover, the beams reinforced with BFRP bars are unlikely to withstand high temperatures for two hours (an example of this type of beam is shown in Figure 5, set 1.1 (sample B2Ø14)). However, it has been demonstrated in previous tests (set 1-prev) that beams reinforced with BFRP bars can withstand high temperatures for an hour, as shown in Figure 4b.

According to the present study (set 1.2), HFRP-RC beams can resist elevated temperatures for two hours. Similar to the previous tests, all samples subjected to residual testing were destroyed due to reinforcement failure in the four-point bending test.

### 5.2. FRP Bars after Applying Elevated Temperatures

**Figure 6:**
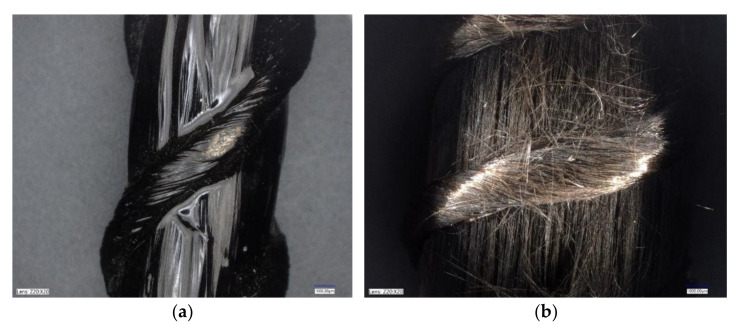
Magnified images of HFRP bars (20 × 20): (**a**) before testing; (**b**) after testing.

The temperature caused burning of the FRP bars, as can be seen in Figure 4 for both BFRP and HFRP bars. This led to the evaporation of the matrix in the middle portion of the bars. Figure 6a shows the surface of the HFRP bar before it was exposed to high temperatures and loads, while Figure 6b shows it after it had been exposed to fire and loads. The bar depicted on Figure 6b is the same uncovered bar as that shown in Figure 4d, indicating that the fibers remained in the same place and continued to withstand the load.

**Figure 7:**
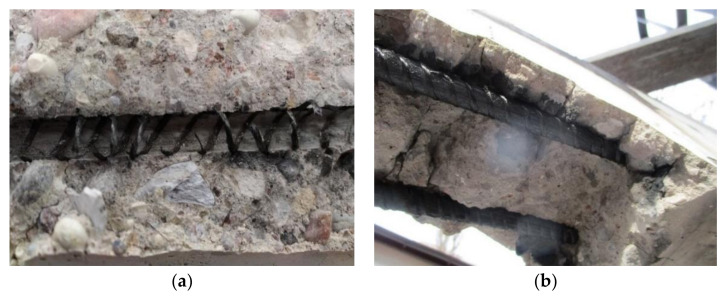
Removal of bars from concrete beams: (**a**) middle part of the beam; (**b**) side part of the beam.

As the sample bars were uncovered from the destroyed elements, it was observed that in the middle part of the beam (where the temperature was applied), the bars were able to be removed easily, in contrast with the side parts. Figure 7a shows that once the glass transition temperature was reached, the matrix evaporated and the contact with the concrete was lost. Consequently, as shown in Figure 7b, the beam side parts were considered as anchors for tested samples.

### 5.3. Temperature Distribution

**Figure 8:**
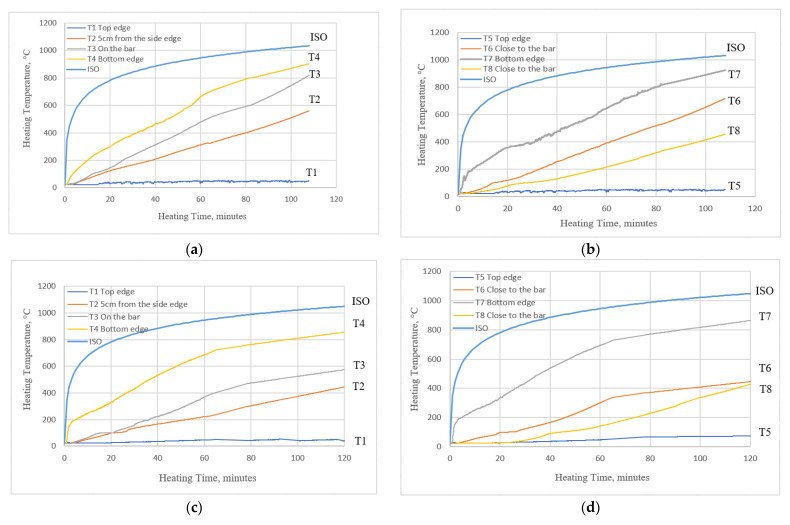
Measurements from the embedded thermocouples: (**a**) B2Ø14, location A; (**b**) B2Ø14, locations B and C; (**c**) H2Ø14, location A; (**d**) H2Ø14, locations B and C.

With the measurements collected from eight thermocouples embedded in the concrete and on the bars, it was possible to analyze the temperatures attained at different depths. The thermocouples were numbered according to the scheme (Figure 2) and corresponded to different locations in the sample. The measurements for one of the samples from set 1.1 (B2Ø14) and one of the samples from set 1.2 (H2Ø14) are displayed in Figure 8.

Through the use of thermocouples, it was observed that the increase in temperature slowed over time, along with the temperature inside the furnace. The outcomes for T4 and T7 for both of the sets were related to the thermocouples at the bottom edge of the beam. For both thermocouples, which recorded similar temperatures, this might indicate that the temperature in the furnace was distributed uniformly. Furthermore, it can be observed that the maximum temperatures obtained at the bottom edge were approximately 20% lower than the applied one (at the end of the heating phase). The measurements obtained from both thermocouples T1 and T5 indicated that ceramic and rock wool insulation were carefully applied to the gaps between the beam interfaces and furnace edges.

Additionally, it can be seen that the results are similar when comparing temperatures measured at thermocouple T3 (on the bar surface) and T6 (nearly the same depth and location). The temperature measured on the bar, however, was slightly higher. The higher temperature may have been because of the burning of the matrix within the bar, as prior to reaching the glass transition temperature there was no significant difference. Thermocouple T3 was located in the midsection of the furnace, whereas thermocouple T7 was located near the side, which could indicate that there were not uniform temperatures inside the beam as there was for the bottom edge of the beam.

Temperatures measured by thermocouple T8 were approximately 30–40% lower than temperatures measured by thermocouple T3. As the glass transition temperature is reached, the bond between the concrete and the bar surface may be compromised in places outside of the furnace but near to it (section C).

### 5.4. Deflections during Heating and Cooling Phases

**Figure 9:**
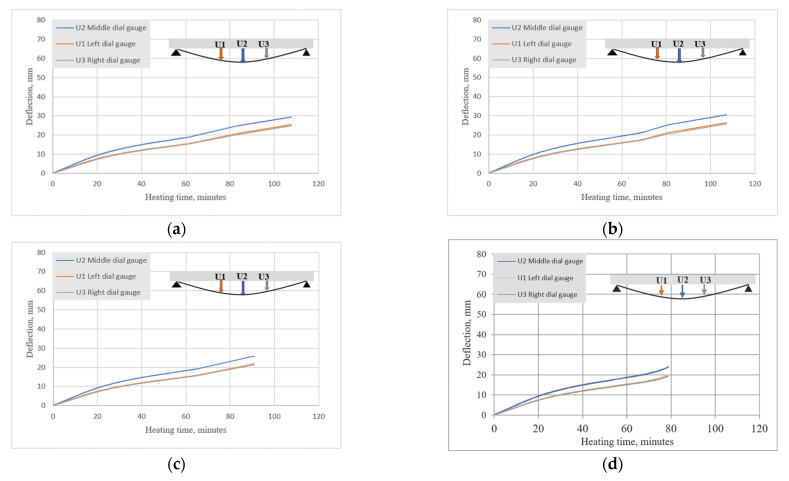
Heating time vs. deflections measured by three dial gauges for set 1.1: (**a**) B2Ø14-beam 1; (b) B2Ø14-beam 2; (**c**) B2Ø14-beam 3; (**d**) set 1-prev: B2Ø14.

The BFRP-RC samples all displayed typical flexural–ductile behavior until failure. Figure 9a–c shows the results of the current study (deflection vs. time) on beams from set 1.1. (B2Ø14), while Figure 9d shows the data for a previous study on a similar beam set 1-prev (B2Ø14). The results confirm the trend seen in previous testing, whereby deflections only increased with elevated temperatures, which is also common for beams reinforced with steel bars [17,18].

For set 1.1, the best recorded result was for B2Ø14, which failed approximately 108 min after heating. In the previous study, BFRP-RC beams were heated for more than one hour. After being exposed to fire, the overall strength capacity of the samples was decreased by approximately 44%. However, since in the current study the beams failed before the end of heating, it was not possible to determine their overall strength capacity. It can be seen that increasing the heating time has a significant impact on the results.

**Figure 10:**
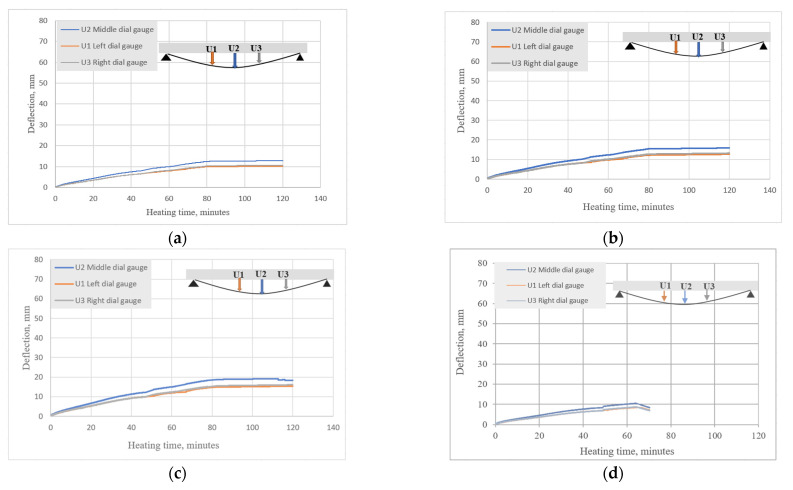
Heating times vs. deflections measured by three dial gauges for set 1.2: (**a**) H2Ø14-sample 1; (**b**) H2Ø14-sample 2; (**c**) H2Ø14-sample 3; (**d**) set 1-prev: H2Ø14.

The HFRP-RC samples (set 1.2) were exposed to elevated temperatures for two hours, and the results proved that they could withstand high temperatures. HFRP-RC beam deflections were measured during the heating phase, which for these beams was equal to 120 min. In Figure 10, it is evident that the HFRP-RC beams displayed atypical behavior; upon reaching a certain temperature, which was approximately 550 °C at approximately 80 min, the deflections began to decrease. As shown in Figure 10d, HFRP-RC beams displayed similar behavior in previous tests (set 1-prev). 

According to the author, this was an important reason why HFRP-RC beams withstood the two-hour period, since the carbon fibers of FRP bars exerted a sort of “prestressing” effect. Smaller beam deflections led to larger depths of compression zones and smaller crack widths. In HFRP-RC beams, thermocouple T4 measurements (Figure 8a,c) on bar surfaces were lower than in BFRP-RC beams, where temperatures penetrated more quickly.

A similar drastic decrease in the deflection of HFRP-RC members was also seen in the previous test (set 1-prev). However, it was not as drastic for the current study. During the pick-up phase in deflections, the remaining fibers were fixed and the deflection started to stabilize. This could be related to the smaller clear cover for the beam assumed in the previous study and the high temperature effect causing the beam to prestress quickly. 

**Figure 11:**
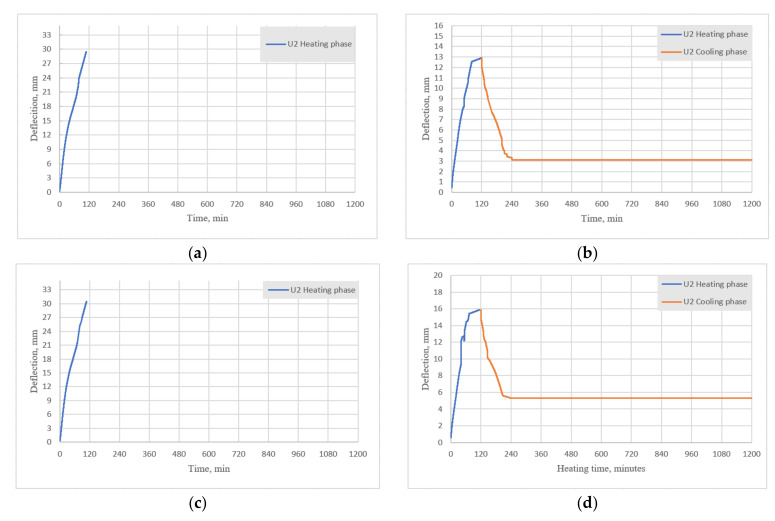

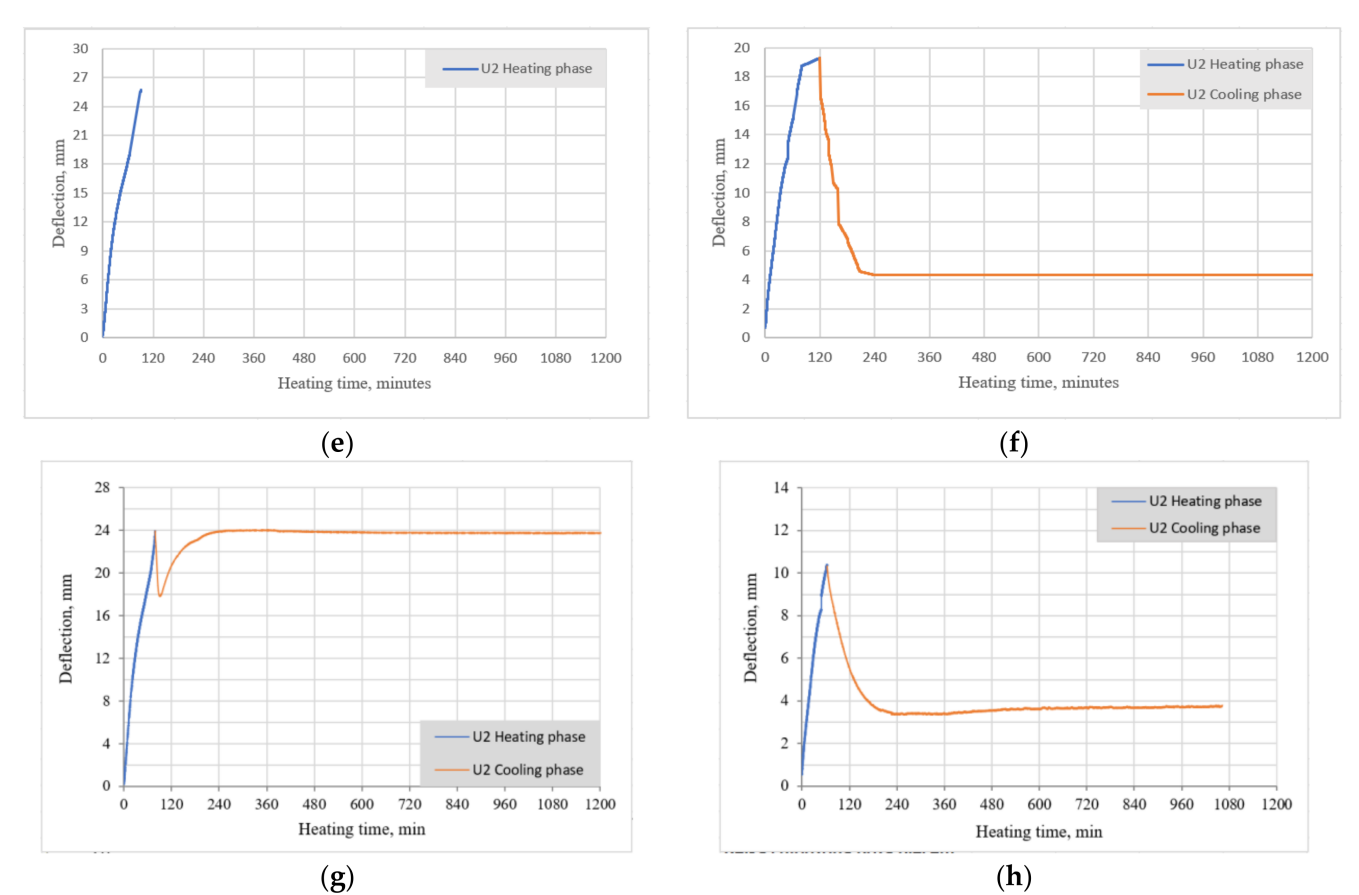
Mid-span deflections measured by U2 dial gauge for (**a**) set 1.1 (B2Ø14-beam 1); (**b**) set 1.2 (H2Ø14-beam 1); (**c**) set 1.1 (B2Ø14-beam 2); (**d**) set 1.2 (H2Ø14-beam 2); (**e**) set 1.1 (B2Ø14-beam 3); (**f**) set 1.2 (H2Ø14-beam 3); (**g**) set 1-prev (B2Ø14); (**h**) set 1-prev (H2Ø14).

In addition, it is important to note how the beams deflected during the cooling phase. Figure 11a,c,e shows the deflections during heating for BFRP-RC beams (set 1.1), Figure 11b,d,f shows the deflections during heating and cooling for HFRP-RC beams in the current study (set 1.2), while Figure 11g,h shows the results of the previous study for B2Ø14 and H2Ø14, respectively.

For both the current and previous tests, the deflections of BFRP-RC beams during the heating phase were approximately two times higher than deflections of HFRP-RC beams. As observed in the previous study (set 1-prev), the deflections of sample B2Ø14 at first started to decrease but then quickly returned to their maximum values; in contrast, the deflections of sample H2Ø14 decreased during the cooling phase until they reached approximately 30% of their maximum value. HFRP-RC reported similar results to the present study and the beam deflections decreased after the first two hours of cooling by approximately 10 mm, then the deflections remained unchanged after that. These results may indicate that the original strength and modulus of the bars can be regained after the bars have been heated to elevated temperatures.

The HFRP-RC samples (set 1.2) remained stable for two hours under elevated temperatures, and then they were tested under four-point bending. The results are compared in the following subsection.

### 5.5. Residual Strength

In the current study, BFRP-RC beams were not tested because the samples did not withstand the effects of elevated temperatures for two hours (set 1.1). Therefore, the strength capacity of the BFRP-RC beams and the results for one BFRP-RC beam from the previous study (set 1-prev) were excluded.

**Figure 12:**
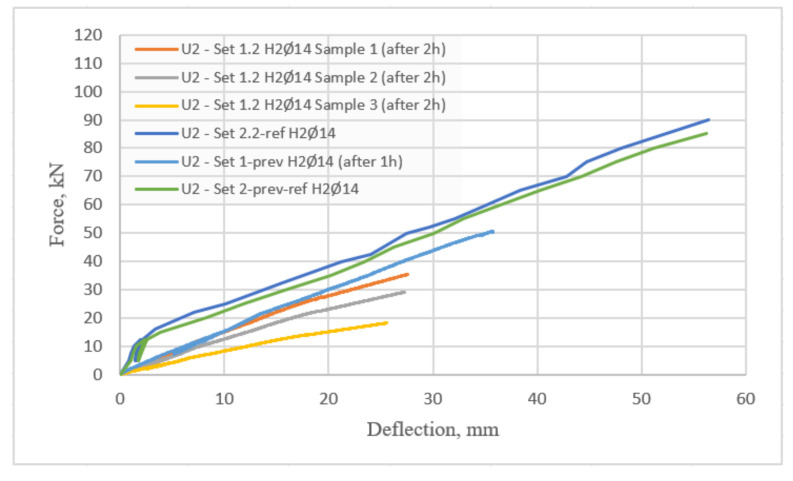
Comparison of ultimate strength capacity values for tested HFRP-RC beams from the current study (set 1.2) with outcomes obtained for reference beams (set 2.2) and results from the previous study (set 1-prev, set 2-prev-ref for HFRP-RC beams).

The ultimate strength capacities of the tested HFRP-RC beams in the current study are compared with results for reference beams and from the previous study in Figure 12. 

The reference beams were only loaded in the four-point flexural tests and had a greater strength capacity. Due to the similarities of the deflection and force curves of the three reference beams in the current study (set 2.2), only one mean curve, which characterizes the behavior of the reference beams, was shown. In addition, the results from a previous study using a beam with slightly different dimensions were quite similar to the results from the current study, as shown in the Figure. The experimental samples were loaded until 12.5 kN and then were reduced to 5 kN, then loaded in one more analogous cycle until failure. The cyclic loading was applied to reduce the effects of plastic strains.

In previous tests where beams were subjected to elevated temperatures for one hour, H2Ø14 samples showed a 40% reduction in strength capacity, as shown in Figure 12. In the current testing, however, three H2Ø14 beams were reduced in strength capacity after two hours of exposure to fire conditions, by 61% for sample 1, 68% for sample 2, and 80% for sample 3. Therefore, the period of heating can be regarded as an important parameter, which may increase the reduction in strength by two-fold.

## 6. Conclusions

HFRP-RC and BFRP-RC beams were examined for residual properties after exposure to elevated temperatures in accordance with the standard heat curve ISO-834. According to the results of the experimental testing, the following conclusions can be drawn:

1. The BFRP-RC samples in the present study did not survive for two hours at elevated temperatures, unlike beams from a previous study, which could withstand higher temperatures for approximately one hour;
2. The strength reduction in HFRP-RC beams after one hour was approximately 40%, while in the present study the strength capacity was reduced by approximately 70%. The above facts may suggest the importance of the heating time parameter;
3. The beams exposed to elevated temperatures were destroyed due to reinforcement failure, whereas the reference beams were destroyed by concrete crushing. This indicates that temperature influences bar strength in a substantial manner;
4. Deflections in BFRP-RC beams were approximately two times greater than those measured for HFRP-RC beams. This can be explained by the presence of carbon fibers in the HFRP bars, which has the effect of “prestressing” HFRP-RC beams, which can be observed during heating and cooling.

According to the author, in light of the observed facts, it is necessary to conduct further analyses comparing various fire scenarios, taking into account simultaneous heating and loading, the influences of different reinforcements on residual strength capability, and other factors. FRP bars can only be implemented in design once a comprehensive study of the behavior of FRP-RC beams during and after fire exposure has been carried out.

## Figures and Tables

**Table 1:** General procedure with regard to considered sets.

Set No.	Description
1.1 and 1.2	In the present study, residual tests were conducted on these beams.
2.1-ref and 2.2-ref	Those beams that were only subjected to flexural tests with four points, without preliminary loads, and that were not subjected to elevated temperatures.
1-prev and 2-prev-ref	The results of that study were used primarily for comparison. Table 2 provides details about the specimens used in the previous study.

**Table 2:** Descriptions of specimens and loading protocols.

Set No.	Beam Designation	Dimensions of the Specimens	Concrete Cover	Number of Samples	Reinforcement Type (Tension Zone)	Preliminary Loaded (Approx. 50% of the Ultimate Load)
		l/h/b ^1^ (mm)	(mm)		Number/Ø/Type	Yes/No (kN)
1.1	B2Ø14	3220/280/140	60 mm from bottom, 40 mm from other sides	3	2/14/BFRP ^2^	Yes (30)
1.2	H2Ø14			3	2/14/HFRP ^3^	Yes (40)
2.1-ref	B2Ø14			3	2/14/BFRP	No (0)
2.2-ref	H2Ø14			3	2/14/HFRP	No (0)
1-prev	B2Ø14	3200/260/140	30 mm from all sides	1	2/14/BFRP	Yes (30)
	H2Ø14			1	2/14/HFRP	Yes (40)
2-prev-ref	B2Ø14			1	2/14/BFRP	No (0)
	H2Ø14			1	2/14/HFRP	No (0)

Note: ^1^ l/h/b refer to length/height/width; ^2^ BFRP means basalt-fiber-reinforced polymers; ^3^ HFRP means hybrid-fiber-reinforced polymers.

**Table 3:** Mechanical characteristics of concrete used for the specimens.

Set No.	Period	Compressive Strength	Tensile Strength	Modulus of Elasticity
		f_c_ (MPa)	f_ct_ (MPa)	E_cm_ (GPa)
1.1; 1.2; 2.1-ref; 2.2-ref	28 days	49.85	4.50	38.91
1-prev; 2-prev-ref		48.75	4.23	37.83

**Table 4:** Mechanical properties of FRP bars.

Type of Bars	Maximum Tensile Force	Tensile Strength	Tensile Strength at Rupture	Modulus of Elasticity
Type/Ø	F_u_ (kN)	f_u_ (MPa)	ε_u_ (%)	E_1_ (GPa)
BFRP Ø6	37.07	1148.81	2.48	46.47
BFRP Ø8	60.03	1103.30	2.52	43.87
BFRP Ø14	179.26	1101.94	2.39	46.02
HFRP Ø14	206.57	1160.06	1.61	72.12

## Data Availability

The data presented in this study are available upon request from the corresponding author.

## Abbreviations

FRP	Fiber-Reinforced Polymers
BFRP	Basalt-Fiber-Reinforced Polymers
HFRP (HC/BFRP)	Hybrid-Fiber-Reinforced Polymers (Hybrid Carbon/Basalt-Fiber-Reinforced Polymers)
GFRP	Glass-Fiber-Reinforced Polymers
CFRP	Carbon-Fiber-Reinforced Polymers
nHFRP	Nano-Hybrid-Fiber-Reinforced Polymers
RC	Reinforced Concrete
FRP-RC	Fiber-Reinforced Polymer Reinforced Concrete
BFRP-RC	Basalt-Fiber-Reinforced Polymer Reinforced Concrete
HFRP-RC	Hybrid Fiber-Reinforced Polymers Reinforced Concrete
DMA	Dynamic mechanical analysis
T_g_	Glass transition temperature
T_d_	Decomposition temperature
B2Ø14	The beams reinforced with 2 bars (BFRP type) of the diameter of 14 mm
H2Ø14	The beams reinforced with 2 bars (HFRP type) of the diameter of 14 mm
T_Iso_	Temperature obtained in accordance with a standard heating curve ISO-834
